# The Bridge Case—Decision of the Court

**Published:** 1896-05

**Authors:** Hoyt H. Wheeler, James C. Chapin, Edwin H. Brown, Charles K. F. Offield


### The Bridge Case—Decision of the Court.

UNITED STATES CIRCUIT COURT, EASTERN DISTRICT OF NEW YORK.

INTERNATIONAL TOOTH CROWN CO.

*vs.*

ALLEN G. BENNETT.

} *In Equity.*

The bill alleges ownership by the plaintiff and infringement by the defendant of patent No. 238,940, dated March 15, 1881, and granted to James E. Low, for a method of permanently fixing artificial teeth to the mouth by bands around the natural teeth, in dentistry. The answer, among other things, denies knowledge, and prays strict proof of ownership; and sets up various anticipations.

At one place a certified copy forms the record of an assignment in the patent office was put in evidence taken on notice, but in absence of defendant's counsel. This is objected to now as insufficient. It would have been inadmissible on objection then; and perhaps have been suppressed on motion afterward (*American Cable Railway Co.* vs. *New York*, 60 Fed. Rep., 1016). But as it has been left as evidence in the case its inadmissibility has been waived; and on that waiver it seems to be sufficient.

The patent was before Wallace and Shipman, J. J., *International Tooth Crown Co.* vs. *Richmond*, in the Circuit Court for the District of Connecticut, 30 Fed. Rep., 775, and sustained. Of course everything decided there is to be considered as settled here.

The method is wholly mechanical, and is said now, in view of *Ridson* vs. *Medart*, 158 U. S., 68, decided since, not to be patentable; and defenses of prior knowledge and use by Doctor Day and by Boctor Beardsley, not before the court then, are relied upon now.

When the method, and not the operating parts, is what is invented, that, of course, is what is to be patented. Here the natural teeth belong to the wearer, and are to be operated upon; they are not made by the inventor to operate, and can not be brought within the patent. The bands were not new in any sense alone; nor were they when combined with the artificial teeth merely; but the mode of attaching the artificial to the natural teeth permanently by the bands might have been; and, if so, that was what was invented, and what should be patented.

This method is thus described in the specifications:

"A band of gold, or other suitable metal, is first prepared and accurately fitted around the tooth adjacent to the vacant spaces to be supplied with an artificial tooth. This band is firmly secured in place by cement, which effectually excludes water or the fluids of the mouth, and is thus permanently attached to the tooth, so that it can not be removed without an operation directly for that purpose. It is sometimes sufficient to prepare one of the adjacent teeth in this way; but generally it is desirable to prepare the adjacent teeth on each side of the vacant space. It will always be advisable to do so if the vacant place is to be occupied with more than one tooth.

"The formation of the mouth and the shapes and position of the teeth are so various with different individuals that my invention may require modification in various particulars in applying it. I, therefore, do not propose to limit myself to the details as shown, but consider that my invention includes the permanent attachment of artificial teeth by securing them to continuous bands permanently attached to adjoining teeth supported upon natural roots, and supporting said artificial teeth by said attachments without dependence upon the gum beneath said artificial teeth."

The claims are for:

"1. The herein described method of inserting and supporting artificial teeth, which consists in attaching said artificial teeth to continuous bands fitted and cemented to the adjoining permanent teeth, whereby said artificial teeth are supported by said permanent teeth without dependence upon the gum beneath.

"2. An artificial tooth cut away at the back, so as not to present any contact with the gum except along its front lower edge, and supported by rigid attachment to one or more adjoining permanent teeth, substantially as and for the purpose set forth."

This method, as such, would be as well practiced and shown by the attachment in that way of one side of one tooth or one end of a block of teeth, to one natural tooth, as by so attaching each side of the single artificial tooth, or each side of the block to a natural tooth. The method of the attachment to a natural tooth is, by the terms of the patent, precisely the same. A band extending upward so as to form a cap over the natural tooth would be none the less a continuing band of the patent when used as such in carrying out this method. The alleged infringement was done only by such use of such cap. Doctor Day testified to soldering a silver cusp to a silver band making a cap, which was permanently attached to a natural tooth of a patient, and to which an artificial tooth was attached. This testimony is supported by that of an assistant learning the profession, that of an intimate acquaintance of the patient, and the production in evidence of the work, kept after long wear.

Doctor Beardsley testifies to making a similar cap of gold and attaching it to a natural tooth of a patient, wife of a clergyman, and to attaching at first an artificial tooth at one side of the cap, and afterward another on the other side, which were worn, and gave satisfaction, several years. In this he is corroborated by an assistant also learning the profession, and by the patient, her two daughters, and one of her Sunday-school scholars. There is nothing so improbable about this testimony which is left wholly undisputed as to leave any fair doubt as to the occurrences, or their date, both of which preceded Low's invention. The method of either seems to be the method of the patent, and either seems to well have anticipated it.

Let a decree be entered dismissing the bill.

JAMES C. CHAPIN, HOYT H. WHEELER.
EDWIN H. BROWN,
For Plaintiff.
CHARLES K. F. OFFIELD,
For Defendant.

